# Entonox^®^ versus Pethidine in Labor Pain Relief: A Randomized Controlled Trial

**DOI:** 10.3390/ijerph182312571

**Published:** 2021-11-29

**Authors:** Rasrawee Chantrasiri, Chanane Wanapirak, Theera Tongsong

**Affiliations:** Department of Obstetrics and Gynecology, Faculty of Medicine, Chiang Mai University, Chiang Mai 50200, Thailand; rcbeautie@gmail.com (R.C.); theera.t@cmu.ac.th (T.T.)

**Keywords:** Entonox^®^, labor pain, meperidine, nitrous oxide, pethidine

## Abstract

Background: Pain relief during labor is a part of standard care in modern obstetrics. Several modalities used for pain relief have their own disadvantages and benefits in terms of side effects, effectiveness, availability, and satisfaction. The objectives of this study are primarily to compare the effectiveness and patients’ satisfaction for pain relief during labor between pethidine and inhaled 50% nitrous oxide (Entonox^®^). Methods: Laboring women at 37–41 + 6 weeks of gestation were randomly allocated to receive pethidine (50 mg intravenously) or Entonox^®^ for reducing labor pain. Pain scores were evaluated at 0, (baseline), 30, 60, 90, and 120 min after initiation, using the visual analog scale (VAS) and also satisfaction score after delivery using the verbal rating scale (VRS). The secondary outcomes were also assessed, including APGAR scores, labor course, side effects, and cesarean section rate. Results: A total of 136 laboring women underwent randomization into two groups, but only 58 and 65 in the pethidine group and the Entonox^®^ group were available for analysis. The median pain scores at baseline, 30, 60, and 90 min were comparable between both groups (*p*-value > 0.05); however, pain score at 120 min in the pethidine group was significantly higher (*p*-value: 0.038). The median of satisfaction score was significantly higher in the Entonox^®^ group (4 vs. 3; *p*-value 0.043). All of the secondary outcomes were comparable between the two groups. Conclusions: Both have comparable effectiveness, but Entonox^®^ has a higher satisfaction score. Entonox^®^ could be an alternative to pethidine for reducing labor pain, because of its efficacy, ease for self-adjustment for satisfaction, and no serious effects on the labor course and newborns.

## 1. Introduction

Labor pain is the most painful experience in a woman’s life. It is caused by myometrium contractions leading to cervical dilatation and effacement. Every pregnant woman must pass through this process before delivering. The severity of pain depends on an individual’s pain threshold, which the more severe pain the more adverse effects on mothers and fetuses.

Several modalities are used to relieve pain during labor including: (1) natural pain relief such as massage, heat, and acupuncture, (2) non-pharmacologic treatment such as water injection for low back pain or transcutaneous electrical neuromuscular stimulation (TENS), and (3) pharmacologic treatment which is commonly used such as intravenous/intramuscular opioids, inhalational analgesics, and epidural blocks. Intravenous pethidine (meperidine) is the most common opioid used for labor pain relief. Pethidine takes effect within 5 min and reaches the maximum effect within 45 min. It is cheap, readily available, and simple to use; however, it can be associated with several side effects such as dysphoria, sedation, respiratory depression, nausea, vomiting and neonatal respiratory depression, which is the most serious side effect. 

Pre-preparation inhaled nitrous oxide (Entonox^®^), containing 50% nitrous oxide and 50% of oxygen, is an inhaled analgesic gas commonly used in general anesthesia. It has been used for labor pain relief since the late 1800s and no serious side effects on both the mother and fetus were reported, although nausea, vomiting, dry mouth, and dizziness may be sometimes encountered. Entonox^®^ has rapid onset, within 30 s and reaches the maximum effect within 2 min. The mechanism of action mainly acts on the central nervous system (CNS) to increase the secretion of endorphins leading to analgesia, euphoria, and a relaxation effect. Surprisingly, in spite of effectiveness and less side effects, nitrous oxide is not so popular and the studies on labor pain relief are limited. Although many studies have been conducted to evaluate the effectiveness of nitrous oxide for pain relief during labor, only few studies were aimed to compare the effectiveness for pain relief during labor between nitrous oxide (Entonox^®^) and meperidine (pethidine). To the best of our knowledge, there is only a study reported by Mobaraki et al. [1], which compared such effects and showed that Entonox^®^ gave better pain relief in the short term when compared to a single dose of pethidine. Systematic review and meta-analysis on nitrous oxide for the management of labor pain reported that only few studies of good or fair quality have been published, and the control groups were very heterogeneous [2]. The review concluded that further research is needed across all of the areas examined: effectiveness, satisfaction, and adverse effects [2,3]. Accordingly, we conducted this study aimed mainly to compare visual analog scores of labor pain and satisfaction levels between women who received nitrous oxide (Entonox^®^) and those who received meperidine, and also to compare the neonatal outcomes, labor courses, and cesarean section rate between both groups.

## 2. Materials and Methods

This study is a randomized controlled trial conducted on Thai pregnant women who were candidates for vaginal delivery at Maharaj Nakorn Chiang Mai hospital, Chiang Mai University, Thailand between August 2020 and April 2021. The study was conducted with ethical approval by the Institutional Review Board (Research ID: FAC-MED-2563-07123). It was registered for clinical control trial study at Thai Clinical Trials Registry (TCTR ID: 20200715007). Written informed consent was obtained from all participants. The study population was pregnant women in labor admitted to our labor room unit. The inclusion criteria were as follows: (1) maternal age between 20 and 40 years; (2) gestational age between 37 and 41 + 6 weeks; (3) singleton pregnancy; (4) vertex presentation; (5) true labor pain, defined as regular uterine contractions (greater than 3 times in 10 min) with progressive change of cervical effacement or dilatation; and (6) no contraindication for vaginal delivery. The exclusion criteria were as follows: (1) pregnancies with medical or obstetric complications such as heart diseases, thyroid diseases, pulmonary diseases (COPD, asthma), infectious diseases, pregnancy-induced hypertension, chronic hypertension, etc.; (2) prior uterine scar or prior cesarean delivery; and (3) contraindications for inhalation analgesia such as pneumothorax or heart failure.

Consecutive women meeting the eligibility criteria were systematically informed of the objective and intervention of the research and were invited to participate. After written informed consents were obtained, the participants were randomly allocated to one of two groups, after they requested a pain relief, using computerized random numbers, with one group using pethidine and the other using Entonox^®^ for labor pain relief. A randomization scheme was prepared by one of the authors (RC) before the study began, and the code for each participant was kept in a sealed, black opaque envelope. Participants of one group were assigned to use Entonox^®^ as a pain reliever, whereas those in the other group were intravenously injected with 50 mg of pethidine. All participants in the Entonox^®^ group were trained for the self-administration of Entonox^®^ by the nurse who was in charge of that case. The participants inhaled gas via a mouthpiece for 30 s before their upcoming uterine contraction.

The participants received the medication on request in the active phase, which was defined as cervical dilatation of ≥5 cm and 100% effacement, or anytime of labor if the pain score was of more than 5 out of 10. The participants received the pain reliever using medications relevant to the group of allocation. The participants in two groups could switch to the other pain control method after 30 min of their initial method if they still had severe pain. The participants in the pethidine group could repeat a pethidine injection at least 4 h after the first dose as our standard guideline.

Before being administered the first dose of pain control, baseline data were prospectively recorded on a standardized form. Baseline pain score, vital signs, interval, and duration of uterine contraction, and fetal heart rate were assessed and recorded every 30 min for a total of 120 min or until delivery in cases where the participants had delivery before 120 min after the first dose. The pain score was determined by using visual analog scale (VAS) ranging from 0 to 10. In case of switching pain control group after 30 min of their initial method at any time point, the pain score would not be further recorded. After delivery, satisfaction score, APGAR score, and route of delivery were recorded. The satisfaction score was evaluated by the verbal rating scale (VRS), which was rated from 1 to 5 (1 = dissatisfied, 2 = mildly satisfied, 3 = moderate satisfied, 4 = satisfied, 5 = extremely satisfied).

The primary outcome measures were medians of the pain scores at each time period and the satisfaction score in the two groups. The secondary outcome measures were the neonatal outcomes based on the APGAR scores and cesarean section rates in the two groups.

Statistical analysis: All of the data were analyzed using the statistical package for the social sciences (SPSS) software version 26.0 (IBM Corp. Released 2019. IBM SPSS Statistics for Windows, Version 26.0 IBM Corp: Armonk, NY, USA). The baseline characteristics were presented as mean+ SD or median (IQR) for continuous data, as percentage for the categorical data. Chi-square, Fisher exact, Mann–Whitney U, and Student’s *t* tests were used for the comparisons as appropriate. A *p*-value of less than 0.05 was considered to be statistically significant. Based on a previous study that demonstrated that a standard deviation of VAS pain score was 1.4 among women receiving Entonox^®^ [1], this study needed a sample size of at least 84 cases, 42 in each group, to gain power of 90% at a 95% confidence interval, when a reduction VAS pain score of 1 was considered clinically significant.

## 3. Results

**Figure 1:**
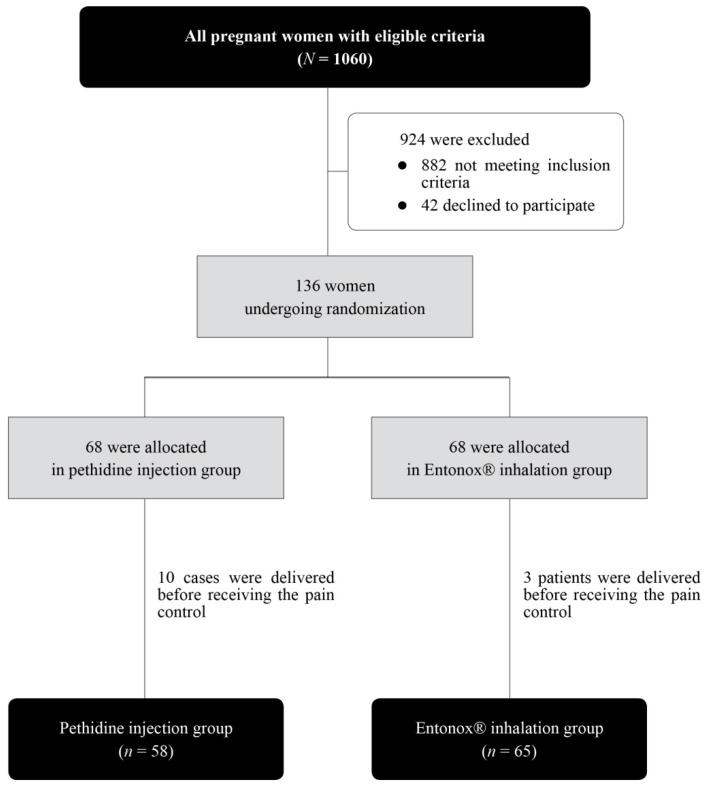
Flow diagram showing enrollment and randomization of the study.

During the study period, a total of 136 pregnant women underwent randomization into two groups: 68 for the pethidine group and 68 for the Entonox^®^ group. However, 10 in the pethidine group and 3 in the Entonox^®^ group had delivery before receiving pain relief. Therefore, 58 and 63 in the pethidine and Entonox^®^ group were respectively available for analysis, as presented in Figure 1.

The baseline characteristics were not significantly different between the two groups, as presented in Table 1.

**Figure 2:**
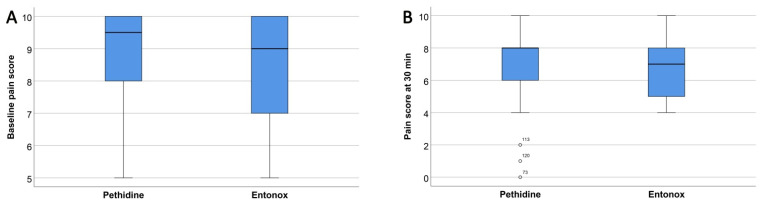

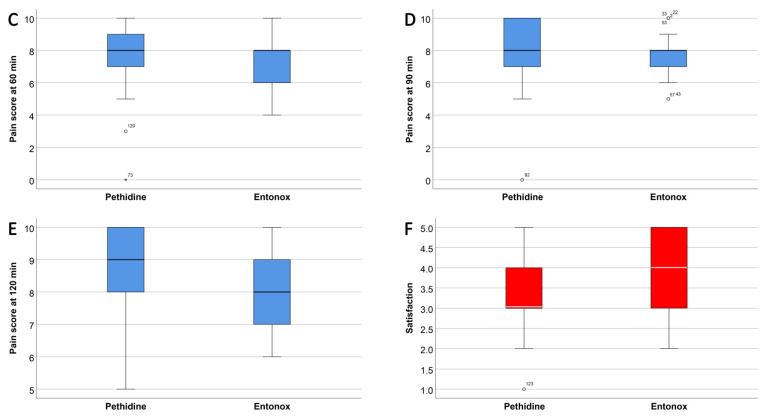
Boxplots comparing pain scores at baseline, 30 min, 60 min, 90 min, and 120 min (**A**–**E**), respectively; as well as satisfaction score (**F**), between both groups.

There was a decrease in median pain score at 30 min of 1.5 points (from 9.5 to 8) and 2 points (from 9 to 7) in the pethidine and Entonox^®^ group, respectively. There were no significant differences of pain scores in both groups at 60 and 90 min of use, as presented in Table 2 and Table 3 and Figure 2. Of note, at 120 min after initiation, the pain score was significantly lower in the Entonox^®^ group (*p*-value: 0.038). Overall, the effectiveness of both techniques for pain relief during labor was comparable. Nevertheless, the median of satisfaction score was significantly higher in the Entonox^®^ group than that in the pethidine group (3 vs. 4, respectively, *p*-value: 0.043), as presented in Table 2 and Figure 2.

In the pethidine group, there were seven participants who required the second dose of pethidine 4 h after the first dose injection and nine participants changed to use Entonox^®^ inhalation at 30 min after injection. In the Entonox^®^ group, seventeen participants required a pethidine rescue dose after 120 min, and nineteen participants changed to use pethidine injection at 30 min after inhalation, as presented in Table 4.

The cesarean section rate was higher in the pethidine group, 22.4% compared with 15.4% in the Entonox^®^ group, but not significantly different, as presented in Table 5.

There were no significant differences reported in both the pethidine and Entonox^®^ group in the interval and duration of uterine contraction, blood pressure, maternal heart rate, and fetal heart rate after 30 min used. Additionally, Apgar scores and stages of labor did not show any significant difference between both groups, as presented in Table 5 and Table 6.

## 4. Discussion

New insight gained from this study is that Entonox^®^ is as effective as pethidine in reducing pain during labor, whereas effects on uterine contractility, length of the first and second stage of labor, cesarean section rate, and Apgar scores are comparable. Nevertheless, pregnant women have significantly higher satisfaction scores in the group of Entonox^®^. Note that although the pain scores at most time line periods were not significantly different between the two groups, the scores in the Entonox^®^ group tended to lower with advanced timeline, and finally, at 120 min, the pain scores in the Entonox^®^ group were significantly lower.

Pethidine is one of the popular medications used for labor pain relief. In addition to well-accepted effectiveness, pethidine is relatively cheap and also available worldwide. One of the already known disadvantages of pethidine is neonatal respiratory depression and often needs opioid receptor antagonists such as naloxone. However, note that our study found no difference in neonatal depression between both groups. This is likely caused by preventive intervention (naloxone) in the pethidine group, which we routinely used as indicated.

Nitrous oxide has been used for labor pain relief since 1800, but surprisingly, it is not so popular, and the number of studies on this issue is very limited. This might be related to less availability in the birthing unit and unfamiliarity of use. In addition, it needs more instruction to use to avoid missed use, causing inadequate inhalation and low efficacy. However, after approval by the US Food and Drug Administration (FDA) in 2012, inexpensive portable nitrous oxide units (delivering a mixture of 50% nitrous oxide and 50% oxygen) become more available as a new option for laboring mothers. Currently, midwives, labor nurses, and physicians are more familiar with this modality, and we expect to witness a resurgence of its use and gain important clinical experience in the role of Entonox^®^ for managing labor pain

Concerning the secondary outcomes, obstetric and neonatal complications, labor course, cesarean section rate, etc. were comparable. However, we cannot conclude that Entonox^®^ has no effect on such problems, since there was no placebo group. However, previous studies showed that rates of pregnant outcomes among the Entonox^®^ group were not significantly different from those in the placebo group [4]. For example, Mobaraki et al. [1] showed that there was no significant difference in maternal and neonatal complications as well as labor duration between the Entonox^®^ group and pethidine group.

To the best of our knowledge, only few studies compared the effectiveness of Entonox^®^ with pethidine [1], while most compared with no intervention, oxygen [5], warm water, spinal analgesia [6], or intramuscular tramadol [7]. Among previous reports, our study is most similar to that of Mobaraki et al. [1] in terms of study design (randomized controlled trial), control (pethidine), and the primary and secondary outcomes. Slightly different, we extended the time length for pain control from 60 min in Mobaraki’s study to 120 min to be more applicable for labor pain and also evaluated patients’ satisfaction. Interestingly, Mobaraki showed Entonox^®^ gave give better pain relief in the short term (30 min) compared to a single dose of pethidine, whereas we found the significant difference in pain reduction only at 120 min of treatment. Nevertheless, the results are in agreement in the effectiveness of Entonox^®^. Based on these two studies, it is reasonable to conclude that Entonox^®^ is as effective as or superior to pethidine in reducing pain in labor, whereas other main effects such as uterine activity and duration of labor are comparable. However, Entonox^®^ is theoretically superior to pethidine in terms of no neonatal respiratory depression and no need to opioid receptor antagonists in newborns.

Note that our results must be interpreted with precaution since, although the difference was statistically significant, the difference was only a small extent, which was possibly not clinically significant. Additionally, this study did not compare pain scores after 30 min of administration because several factors were involved after this period. We allowed the patients to feel free for switching the methods. Of note, the number of patients who switched from the Entonox group to the pethidine group was higher than vice versa. This seems to be favorable for pethidine but actually this did not mean that pethidine could relieve pain better than Entonox^®^ after 120 min of administration, since pain score was not assessed and the effectiveness could be confounded by many factors such as more pronounced pain in late labor, patient exhaustion, a higher cost of Entonox. Several patients might try to have another method to see if it was better or not, while whether the scores were better or worse than the previous method was not evaluated. Some might prefer to switch to pethidine because it was viewed as a conventional or standard treatment (pethidine) in our daily practice. The results of comparison could be reliably evaluated only in the first 30 min of administration. However, based on the primary results, it is reasonable to conclude that Entonox can clinically be an option for labor pain relief because its effectiveness is comparable or possibly superior to pethidine.

The strengths of this study are as follows: (1) as the nature of randomized controlled trials, known and unknown confounding factors were expected to equally distribute to both groups, as documented by the comparable baseline data; (2) several obstetric and neonatal outcomes were also assessed to determine the effects of both modalities on other than pain relief.

The limitations of this study may include the following. (1) Due to obvious differences in administration techniques, the patient group could not be blinded to both patients and doctors, possibly leading to a bias in the subjective evaluation of pain scoring. (2) The sample might be relatively small for some secondary outcomes without enough power to express a significant effect, if it existed, such as neonatal depression. (3) Since the time of evaluation was confined to only 120 min, the result interpretation might not reflect the overall true effect of the medications and possible overestimation of the effect of Entonox^®^. (4) As seen in Table 2, the sample size is relatively small for comparisons at 120 min.

While the effectiveness of Entonox^®^ and pethidine is comparable, this study favorably supports the use of Entonox^®^ for pain relief during labor because of several advantages such as simplicity of use, world-wide availability, ease for self-adjustment for satisfaction, and no serious effects on the newborns. Additionally, Entonox^®^ could be used as a bridge to neuraxial analgesia or a natural childbirth. Many women start labor unsure about whether they need the use of neuraxial analgesia. Of them, Entonox^®^ may be an option for pain relief during labor, thus giving them more time to make a decision about whether to have regional analgesia. With this approach, several women who use Entonox^®^ early in labor subsequently request neuraxial analgesia, while several women use Entonox^®^ for labor pain relief throughout labor course. Finally, Entonox^®^ may also have a role for reducing postpartum pain. In some women, postpartum complications need surgical procedures, such as repair of perineal lacerations or manual removal of the placenta. Entonox^®^ can facilitate quick completion of the procedures. The use of Entonox^®^ for laboring women may be modified to reduce pain in various situations. For examples, Entonox^®^ may be used as an adjunct to pethidine to potentiate pain relief. Attar et al. [8] conducted a double-blind randomized clinical trial to compare the analgesic effects of Entonox^®^ during labor on reducing the need for pethidine and fetal–maternal complications. They demonstrated that Entonox^®^ significantly reduced pain during labor without significant increase in maternal and neonatal complications.

## 5. Conclusions

Entonox^®^ could be an alternative to pethidine for reducing labor pain because of its efficacy, ease for self-adjustment for satisfaction, and no serious effects on the labor course and newborns. Clinically, this study, together with previous studies, offers pregnant women more options to choose the techniques of pain relief during labor that are most suitable for themselves. Finally, since there are a very limited number of the studies on comparing the effectiveness of the two methods, this study can serve as a source for future systematic review and meta-analysis.

## Figures and Tables

**Table 1:** Comparisons of baseline maternal characteristics between pethidine injection group and Entonox^®^ inhalation group.

Characteristics	Pethidine Injection Group (*n* = 58)	Entonox^®^ Inhalation Group (*n* = 65)	*p* Value
Age (year): mean ± SD	29.0 ± 5.4	29.3 ± 4.2	0.712
Parity Nulliparous Multiparous	43 (74.1%) 15 (25.9%)	47 (72.3%) 18 (27.7%)	0.819
Gestational age 37–39 + 6 weeks 40–41 + 6 weeks	45 (77.6%) 13 (22.4%)	56 (86.2%) 9 (13.8%)	0.216
Gestational age (day): mean ± SD	274.5 ± 6.7	273.7 ± 6.3	0.193
Birth weight: mean ± SD	3155 ± 341	3143 + 379	0.726

**Table 2:** Comparisons of pain scores and satisfaction score between the pethidine group and Entonox^®^ group.

Pain Scores and Satisfaction Score	Pethidine Group: (*n* = 58) Median Score (IQR)	Entonox^®^ Group: (*n* = 65) Median Score (IQR)	*p* Value *
Pain scores			
• Baseline	9.5 (8.0–10.0) (*n* = 58)	9.0 (7.0–10.0) (*n* = 65)	0.130
• At 30 min	8.0 (6.0–8.0) (*n* = 58)	7.0 (5.0–8) (*n* = 65)	0.608
• At 60 min	8.0 (7.0–9.0) (*n* = 51)	8.0 (6.0–8.0) (*n* = 54)	0.151
• At 90 min	8.0 (7.0–10) (*n* = 39)	8.0 (6.5–8.5) (*n* = 42)	0.106
• At 120 min	9.0 (8.0–10.0) (*n* = 34)	8.0 (6.5–9.0) (*n* = 29)	0.038
Satisfaction score	3.0 (3.0–4.0) (*n* = 58)	4.0 (3.0–4.5) (*n* = 65)	0.043

* Mann–Whitney *U*-test.

**Table 3:** Mean difference of pain score in pethidine injection group and Entonox^®^ inhalation group.

Pain Score Difference	Pethidine Injection Group (*n* = 58)	Entonox^®^ Inhalation Group (*n* = 65)	*p*-Value
Baseline and 30 min	−1.5 ± 1.7	−1.1 ± 1.8	0.260
• At 30 min and 60 min	0.8 ± 1.1	0.67 ± 1.3	0.448
• At 60 min and 90 min	0.4 ± 1.9	0.6 ± 1.0	0.647
• At 90 min and 120 min	0.6 ± 1.7	0.5 ± 0.9	0.946
Baseline and 60 min	−0.8 ± 1.9	−0.5 ± 1.7	0.524
Baseline and 90 min	−0.7 ± 2.1	−0.2 ± 1.6	0.259
Baseline and 120 min	−0.2 ± 1.3	0.4 ± 1.6	0.112

**Table 4:** Additional dose of pethidine injection and switching group data.

	Pethidine Injection Group (*n* = 58)	Entonox^®^ Inhalation Group (*n* = 65)	*p*-Value *
Received additional dosage of pethidine (pethidine rescue)	7 (12.1%)	17 (26.2%)	0.049
Shift to another group	9 (32.1)	19 (67.9)	<0.001

* Mann-Whitney *U* test.

**Table 5:** Comparisons of Apgar score and route of delivery between pethidine group and Entonox^®^ group.

Outcomes	Pethidine Injection Group (*n* = 58)	Entonox^®^ Inhalation Group (*n* = 65)	*p*-Value
APGAR score			
• At 1 min: Median (IQR)	9.0 (8.0–9.0)	9.0 (8.0–9.0)	0.731 *
• At 5 min: Median (IQR)	10.0 (9.0–10.0)	10.0 (9.0–10.0)	0.462 *
• At 10 min: Median (IQR)	10.0 (10.0–10.0)	10.0 (10.0–10.0)	0.386 *
Route of delivery, *n* (%)			0.515 #
• Normal delivery	41 (70.7)	46 (70.8)	
• Vacuum extraction	3 (5.2)	6 (9.2)	
• Forceps extraction	1 (1.7)	3 (4.6)	
• Cesarean section	13 (22.4)	10 (15.4)	

* Mann-Whitney *U* test; # Chi-square test.

**Table 6:** Comparisons of other secondary outcomes in pethidine group and Entonox^®^ group.

Secondary Outcomes	Pethidine Injection Group	Entonox^®^ Inhalation Group	*p*-Value *
Uterine contraction	(*n* = 58)	(*n* = 65)	
Interval; minute ± SD Before After 30 min	4.37 ± 2.43 3.12 ± 1.06	4.36 ± 2.02 3.28 ± 1.14	0.957 0.411
Duration; second ± SD Before After 30 min	43.97 ± 8.59 48.58 ± 7.01	47.23 ± 8.61 50.58 ± 5.94	0.038 0.090
Blood pressure; mean ± SD	(*n* = 58)	(*n* = 65)	
SBP (mmHg) Before After 30 min	120.29 ± 16.43 120.60 ± 9.60	120.20 ± 8.13 121.00 ± 9.33	0.968 0.817
DBP (mmHg) Before After 30 min	80.36 ± 8.52 75.17 ± 8.45	77.74 ± 6.49 76.49 ± 7.94	0.056 0.374
Pulse rate (bpm)	(*n* = 58)	(*n* = 65)	
Before After 30 min	85.10 ± 16.15 80.66 ± 10.92	84.69 ± 12.97 80.08 ± 11.86	0.876 0.780
Fetal heart rate (bpm)	(*n* = 58)	(*n* = 65)	
Before After 30 min	141.97 ± 7.63 136.18 ± 11.11	144.68 ± 7.47 139.63 ± 8.68	0.049 0.056
Stage of labor minute ± SD	(*n* = 45)	(*n* = 55)	
First stage of labor Second stage of labor Third stage of labor	688.22 ± 648.54 22.93 ± 15.29 6.67 ± 3.72	706.85 ± 528.63 28.31 ± 24.95 6.11 ± 3.59	0.875 0.209 0.450

* Student *T* test.

## Data Availability

The datasets analyzed during the current study are available from the corresponding author upon reasonable request.
